# Adaptability and Evolution of Gobiidae: A Genetic Exploration

**DOI:** 10.3390/ani12141741

**Published:** 2022-07-06

**Authors:** Yongquan Shang, Xibao Wang, Gang Liu, Xiaoyang Wu, Qinguo Wei, Guolei Sun, Xuesong Mei, Yuehuan Dong, Weilai Sha, Honghai Zhang

**Affiliations:** College of Life Sciences, Qufu Normal University, Jingxuan West Street No. 57, Qufu 273165, China; yongquanshang@163.com (Y.S.); wangxibao1995@163.com (X.W.); liugang970219@126.com (G.L.); wuxiaoyang1988@126.com (X.W.); qgwei2008@163.com (Q.W.); sunguolei1989@163.com (G.S.); meixsunique@126.com (X.M.); dongyuehuan2019@163.com (Y.D.); shaweilai@163.com (W.S.)

**Keywords:** Gobiidae, evolution, mitogenome, positive selection, adaptation

## Abstract

**Simple Summary:**

Animals living in different environments must overcome different environmental pressures. Previous studies have explored this phenomenon for other aquatic organisms, including *Fundulus* genus, intertidal spiders, and intertidal chitons. Gobiidae is a group of bony fishes that is second most diverse group of vertebrates globally, and one of the most diverse families of fish. These fish occupy different environments, including marine, brackish, and freshwater habitats. One key reason for the successful colonization of different habitats by this group is their ability to adapt to different energy demands. Energy requirement is related to the ability of mitochondria in cells to generate energy via a process called oxidative phosphorylation (OXPHOS). This study explored the genetic mechanisms underlying the adaptability of Gobiidae to different environments and energy requirements and, hence, their evolution.

**Abstract:**

The Gobiidae family occupy one of the most diverse habitat ranges of all fishes. One key reason for their successful colonization of different habitats is their ability to adapt to different energy demands. This energy requirement is related to the ability of mitochondria in cells to generate energy via oxidative phosphorylation (OXPHOS). Here, we assembled three complete mitochondrial genomes of *Rhinogobius shennongensis*, *Rhinogobius wuyanlingensis*, and *Chaenogobius annularis.* These mitogenomes are circular and include 13 protein-coding genes (PCGs), two rRNAs, 22 tRNAs, and one non-coding control region (CR). We used comparative mitochondrial DNA (mtDNA) genome and selection pressure analyses to explore the structure and evolutionary rates of Gobiidae mitogenomics in different environments. The CmC model showed that the *ω* ratios of all mtDNA PCGs were <1, and that the evolutionary rate of *adenosine triphosphate 8* (*atp8*) was faster in Gobiidae than in other mitochondrial DNA PCGs. We also found evidence of positive selection for several sites of *NADH dehydrogenase* (*nd*) *6* and *atp8* genes. Thus, divergent mechanisms appear to underlie the evolution of mtDNA PCGs, which might explain the ability of Gobiidae to adapt to diverse environments. Our study provides new insights on the adaptive evolution of Gobiidae mtDNA genome and molecular mechanisms of OXPHOS.

## 1. Introduction

Gobiidae is the second most diverse group of vertebrates, and is one of the most diverse families of fish. This family consists of more than 210 genera and over 1950 described species (i.e., nearly 10% of all fish species), with many species yet to be described [1]. The fish of this family occupy a wide range of environments, including marine, brackish, and freshwater habitats [2,3]. Gobiidae require different amounts of energy to survive in different types of habitats. Mitochondria, which are organelles found in eukaryotic cells [4], are the main site of aerobic cell respiration, converting organic matter into energy, and are the energy factories of cells [5]. Vertebrate mitochondria are double-stranded molecules that have closed structures, usually about 14 to 16 kB in size [6]. The two strands of mitochondrial DNA are: (1) a guanine-rich strand, known as the heavy (H) strand and (2) a cytosine-rich light (L) strand [7]. Mitochondria consist of 13 protein-coding genes (PCGs), 22 tRNAs (transfer RNA) genes, two rRNAs (ribosomal RNA) genes, and one D-loop (control region) [8,9,10,11]. Mitochondrial stress has a large impact on the metabolic performance of an organism [12]. Maintaining mitochondrial integrity and the signals contained therein are essential for cellular homeostasis and survival [13,14]. Mitochondrial genes have been widely used to elucidate phylogenetic relationships among species because of their simple structure, rapid evolution rate, maternal heredity, and minimal recombination [15,16,17]. Therefore, the mitogenome is widely used for the molecular identification, evolution, and genetic relationships of inter-organism systems [18,19]. Estimation of selection pressures acting on mitochondria could provide deep insights on the adaptive evolution of the mitochondrial genome.

Previous studies have explored characteristics of mitochondrial genes of some aquatic organisms [8,20,21]. One study found that the mitochondrial genes of *killifish* in the genus *Fundulus*, occupying different environments evolved at variable rates, but exhibited very low *dN/dS* ratios across all lineages of the genus [22]. Li et al. (2021) constructed a novel mitogenome for the intertidal spider (*Desis jiaxiangi*) and found that positive selection signals in the mitogenome might adapt to different environments [23]. Dhar et al. (2020) observed positive selection in mtDNA PCGs of intertidal chitons (Mollusca: Polyplacophora), indicating that mtDNA PCGs have a role in metabolic adaptations to different environments [12]. To date (April 2022), there have been no reports of mitogenome assemblages in Gobiidae occupying different habitats. Given the critical role of mitochondria in aerobic respiration and adaptation of organisms to different environments, here, we explored the adaptive evolution and phylogenetic relationships of Gobiidae mitogenomes. Our findings are expected to provide new insights on the molecular mechanisms responsible for the adaptation (to different environments) and evolution of Gobiidae.

We assembled and annotated three new complete Gobiidae mitogenomics (*Chaenogobius annularis, Rhinogobius shennongensis, Rhinogobius wuyanlingensis*) based on high-quality whole mitochondrial genomes. To explore the evolution of Gobiidae, we combined data from 18 publicly available mitogenomes. All species were divided into three groups according to their habitat: freshwater group (FG), seawater group (SG), and euryhaline group (EG). We constructed a phylogenetic tree of 21 species using the Bayesian method, and studied their genome structure and selection. Our study provides new mitogenomic resources and enhances our understanding of the molecular mechanisms underlying the adaptation to the different environments and evolution of Gobiidae.

## 2. Materials and Methods

### 2.1. Assembly and Annotation of the Complete Mitogenome

Based on the raw data of Gobiidae genome sequencing (*Rhinogobius shennongensis*, SRR18029980; *Rhinogobius wuyanlingensis*, SRR18030029; *Chaenogobius annularis*, DRR174911.1) that we obtained from the NCBI database, we assembled the three complete mitochondrial genomes using NOVOPlasty 4.1 [24]. The seed sequence was MK758112.1 (*Myersina filifer*). We used BLAST to validate and revise the assembled sequences based on genome data, seed sequences, and MITOS web (http://mitos.bioinf.uni-leipzig.de/index.py (accessed on 24 February 2022) to annotate the mitochondrial genome [25]. The results of the annotation were compared with annotations of mitochondrial genomes of related species (*Myersina filifer* and *Gymnogobius urotaenia*) using BLAST. The three complete mitogenomes were submitted to GenBank under the accession numbers OM830225 (*Chaenogobius annularis*), OM961050 (*Rhinogobius shennongensis*), and OM961051 (*Rhinogobius wuyanlingensis*). A map of the three new complete mitogenomics was generated using the MPI-MP CHLOROBOX website (https://chlorobox.mpimp-golm.mpg.de/geseq.html (accessed on 27 February 2022). MEGA X [26] was used to calculate the relative synonymous codon usage (RSCU) of the mtDNA PCGs. Codon W was used to determine the effective number of codons (ENc) and the GC content of the codons 3rd position option (GC3s) [27]. Comparative alignments of the 21 mitogenomes were performed using Mauve software (2.3.1). Comparative codon usage among the 21 selected mitogenomes was represented by heatmaps using R software.

The three species (i.e., OM830225, OM961050, and OM961051) were divided into three groups according to their habitats: freshwater (FG), seawater (SG), and euryhaline (EG) (Table 1). The mitochondrial genome accession numbers of Gobiidae are presented in Table 1. The composition skew values were calculated according to the following formulas: AT skew [(A − T)/(A + T)] and GC skew [(G − C)/(G + C)] [28]. 

### 2.2. Phylogenomic Analyses

We performed phylogenetic analyses of 21 mitogenomic sequences based on 13 mtDNA PCGs (*nd1*, *nd2*, *co1*, *co2*, *atp8*, *atp6*, *co3*, *nd3*, *nd4l*, *nd4*, *nd5*, *nd6*, *cyb*). We used MUSCLE v3.8.31 to align 13 mtDNA PCGs [29]. Bayesian inference (BI) was used to infer phylogenetic relationships. We used the model finder function of the PhyloSuite software [30] to select the optimal model (GTI + T + G).

Phylogenetic status was determined by comparing the combined mitochondrial gene set (13 mtDNA PCGs and two rRNA genes) based on Bayesian inference (BI). BI was performed using MrBayes [31] with four simultaneous Markov Chain Monte Carlo (MCMC) chains, running for 2,000,000 cycles and sampling every 1000 generations. The Interactive Tree of Life (ITOL) website [32] was used to visualize the derived BI trees. 

### 2.3. Selection Analyses

Evolutionary constraints on individual PCGs in terms of selection pressure were estimated by the ratio of the non-synonymous substitution rate to synonymous substitution rate (*ω* = *dN/dS*) using PAML 4.9j [33]. The selected branch model was the free-ratio model (model = 1, NS sites = 0), which assumes different branches have different *ω* values, but independent values. To evaluate selective pressure, the *ω* values of 13 mtDNA PCGs and each mtDNA PCG of 21 species were calculated. If the *ω* value was equal to zero, we assigned the numerical value as “NA” in the *ω* value dataset (Supplementary Table S2). We used one-way ANOVA and multiple comparison analyses to compare the ω values (a value calculated from a concatenated sequence of 13 mtDNA PCGs and 13 values calculated from 13 mtDNA PCGs). The *p*-values were corrected using the false discovery rate (FDR) [23]. The *ω* values of 1, <1, or >1 in protein-coding sequences may be interpreted as neutral mutation, negative (purifying) selection, or positive (diversifying) selection, respectively. We used R 4.0.3 software to draw a boxplot of the *ω* values. To evaluate the evolutionary rates of Gobiidae in the three environments, we used CmC model analyses. We used clade model C (CmC, model = 3, NSsites = 2, ncatG = 3; null model, M2a_rel, model = 0, NSsites = 22) to compare the evolutionary rates of each mtDNA PCG in response to the three environments. To determine whether positive selection of the 13 mtDNA PCGs occurred in groups differentiated by environment and survival pressures, the EG groups were used as foreground branches, and a branch-site model was used for the analysis. In all analyses (Table S1), the BI tree file was used as the input file for PAML.

## 3. Results

### 3.1. Mitogenome Organization

**Figure 1:**
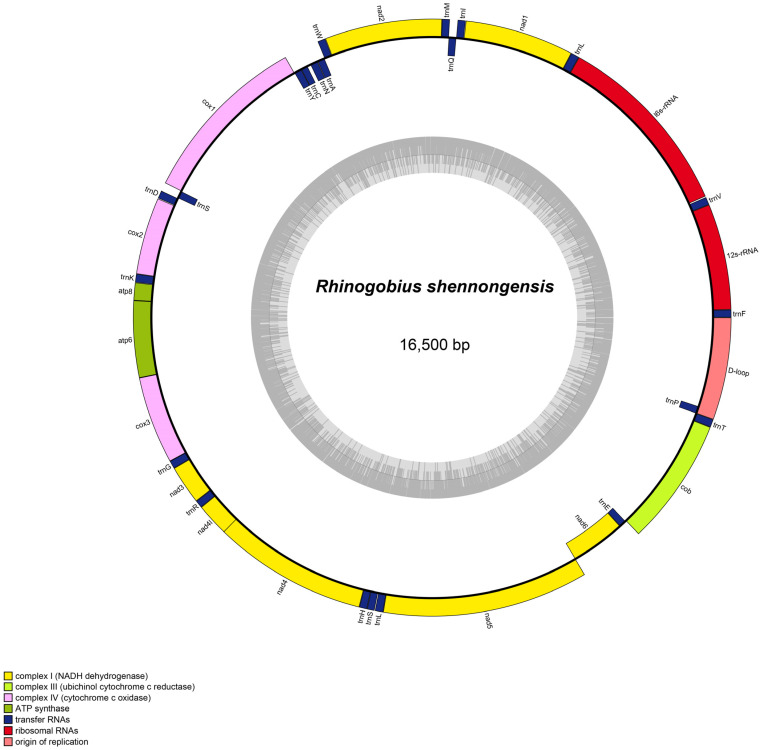
Gene map of mitogenome of *Chaenogobius annularis*. The genes outside the circle are transcribed clockwise, whereas the genes inside the circle are transcribed counterclockwise.

The complete mitogenomes of *Chaenogobius annularis*, *Rhinogobius shennongensis*, and *Rhinogobius wuyanlingensis* were assembled, and were 16,477 bp, 16,500 bp, and 16,491 bp in size, respectively. The complete mitogenome genes contained 13 PCGs, 22 tRNA genes, and two rRNA genes (Figure 1 and Figure S1). It also has a major non-coding region (D-loop) (Figure 1 and Figure S1). The position of each gene in the mitogenome was identical to that in other Gobiidae species. One of the 13 PCGs (*nad6*) and eight tRNAs (trnE, trnP, trnY, trnC, trnN, trnA, trnQ, and trnS2) were encoded by the light strand, whereas the other 28 genes were encoded by the heavy strand (Table 2, Table 3 and Table 4). There were 22 bp, 27 bp, and 24 bp overlaps across the complete mitogenomes of *Chaenogobius annularis*, *Rhinogobius shennongensis* and *Rhinogobius wuyanlingensis*, respectively. In particular, 7 bp had the greatest overlap between *atp8* and *atp6*, and *nd4l* and *nd4*.

**Figure 2:**
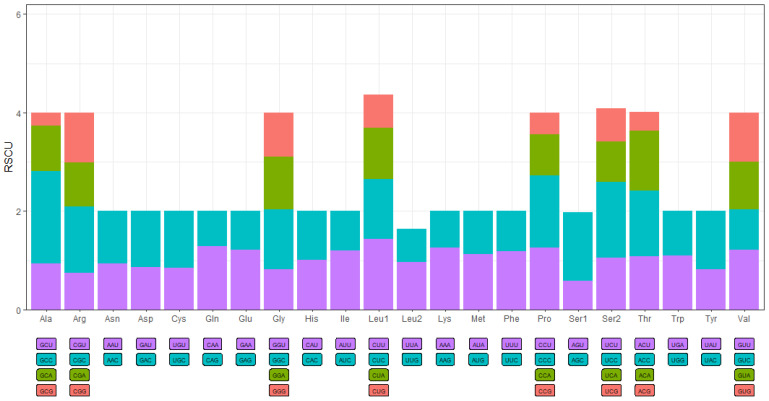
Relative synonymous codon usage (RSCU) of *Chaenogobius annularis*. Codon families are plotted on the x-axis. Codon type is presented beneath each codon family. On the histogram, the proportion of each codon type (retaining same color code) as a proportion of the respective codon family.

The nucleotide composition of the complete mitogenome was delineated (Table 1). Specifically, the GC-content of the four heavy strands was 41.1% (*Chaenogobius annularis*), 47.7% (*Rhinogobius shennongensis*), and 46.8% (*Rhinogobius wuyanlingensis*). The AT content of the entire heavy strand (*Chaenogobius annularis*, 58.9%; *Rhinogobius shennongensis*, 52.3%; and *Rhinogobius wuyanlingensis*, 53.2%) was distinctly higher than the GC content. The overall AT-skew and GC-skew in the whole mitogenome of *Chaenogobius annularis*, *Rhinogobius shennongensis*, *Rhinogobius wuyanlingensis* were 0.145 and −0.204, 0.044 and −0.312, and 0.070 and −0.306, respectively (Table 4). The AT skew for the whole mitogenome, except for the D-loop, was slightly positive, whereas the GC skew was slightly negative. This result implies that A has a higher occurrence than T, while C has a higher occurrence than G. In the mitogenome of *Chaenogobius annularis*, *Rhinogobius shennongensis*, and *Rhinogobius wuyanlingensis*, the initiation codon of mtDNA PCGs was GTG (numbers = 1) and ATG (12). Conversely, the termination codons of *Chaenogobius annularis* were TAG (3), TAA (6), TA (1), and T (3) (Table 2). The termination codons of *Rhinogobius shennongensis* were TAG (1), TAA (7), TA (1), and T (4) (Table 3). The termination codons of *Rhinogobius wuyanlingensis* were TAG (2), TAA (6), TA (1), and T (4) (Table 4). Based on the codon usage analysis used, the eight codon families (Ala, Arg, Gly, Leu1, Pro, Ser2, Thr, and Val) exhibited a strong preference for the three species (Figure 2 and Figure S2).

### 3.2. Comparison of Mitogenomes among Species

**Figure 3:**
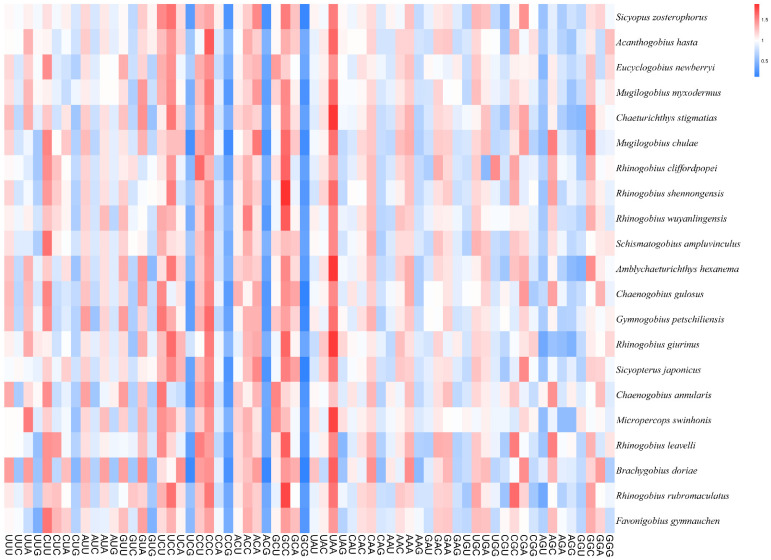
Heatmap based on codon usage of the 21 species evaluated in this study.

**Figure 4:**
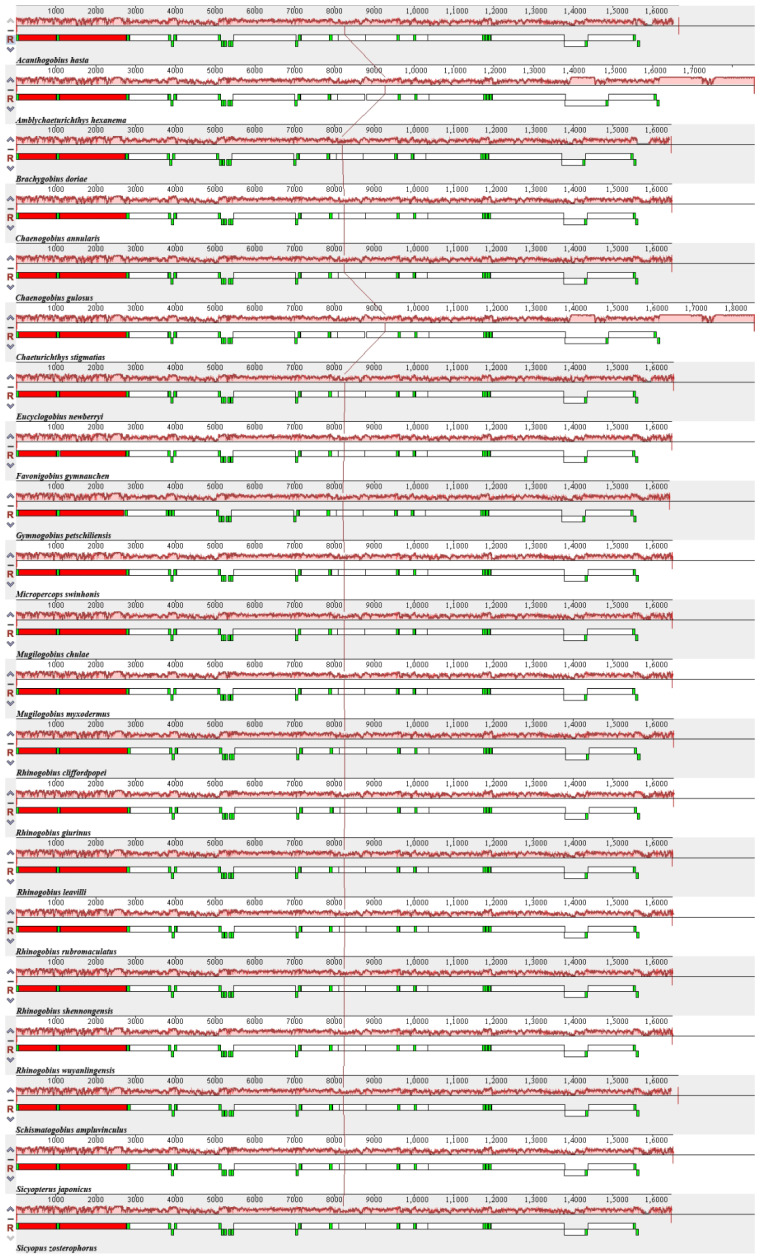
Alignment of the 21 species evaluated in this study using Mauve. Gene arrangement is shown in Table 2, Table 3 and Table 4. Red block is 12S rRNA and 16S rRNA, green block is tRNA, and white block is PCG.

**Figure 5:**
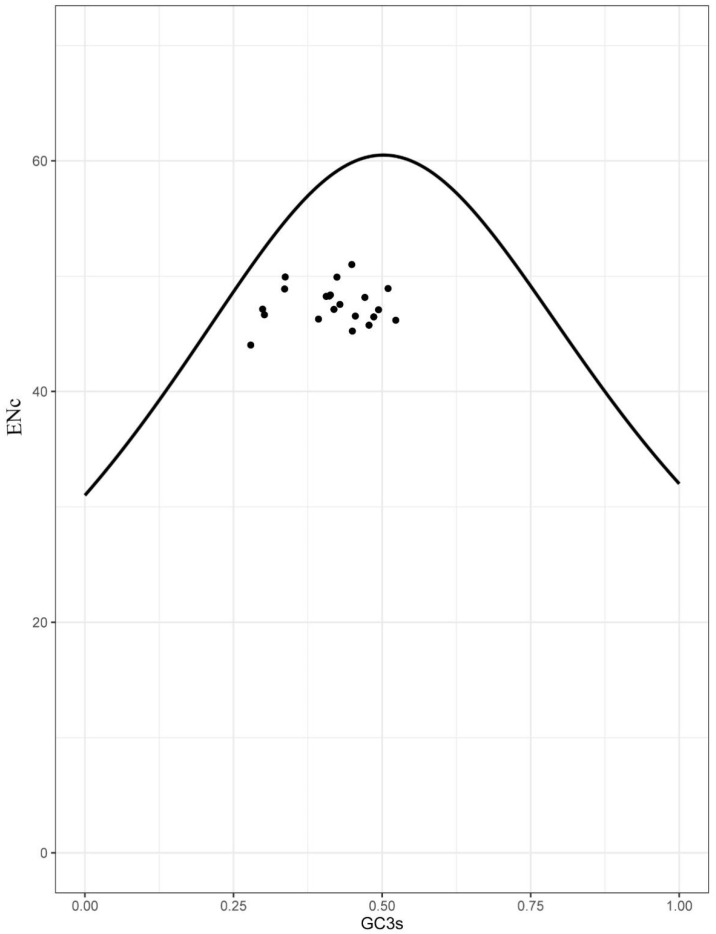
ENc vs. GC3 plot showed that the analyzed mitogenomes were translationally efficient and that natural selection played a crucial role in their evolution.

Heatmaps based on codon usage (Figure 3) showed that CUU (L), UCU (S), UCC (S), CCC (P), and GCC (A) were the most frequently used codons in the selected mitogenomes. Comparative alignments of the 21 mitogenomes were carried out in Mauve, which showed that the gene order (or synteny of these mitogenomes) was, to a great extent, conservative (Figure 4). An umbrella line was drawn for the “expected ENc value” (i.e., value based on the assumption that only mutational pressure acts on the considered genes), and was compared to the observed ENc values (Figure 5). The effective number of codons (ENc) revealed that all investigated mitogenomes were well below the selection pressure curve.

### 3.3. Phylogenetic Analysis

**Figure 6:**
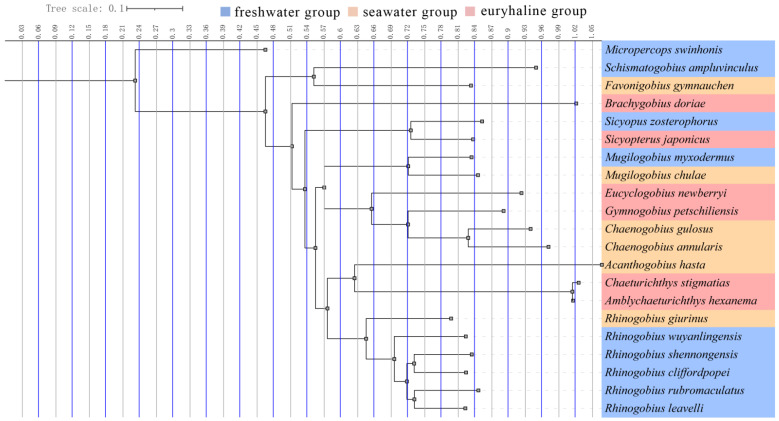
BI tree of all 21 species evaluated in this study based on the 13 mtDNA PCG dataset. Different colors represent different habitat types.

All Gobiidae species were clustered in a well-supported clade with high bootstrap support values and Bayesian posterior probabilities (Figure 6). *Chaenogobius annularis* and *Chaenogobius gulosus* converged in the same clade, whereas *Rhinogobius shennongensis*, *Rhinogobius wuyanlingensis*, *Rhinogobius rubromaculatus*, *Rhinogobius leavelli*, *Rhinogobius cliffordpopei*, and *Rhinogobius giurinus* converged in a separate clade. These evolutionary relationships were consistent with traditional morphological classifications and previous studies [1,34,35].

### 3.4. Selection Analyses

The *ω* (*dN/dS*) values from the 13 mtDNA PCGs and each mtDNA PCG of the mitogenomes of 21 species were estimated using the free-ratio model in the PAML package. All *ω* values (13 mtDNA PCGs and each mtDNA PCG of 21 species) were <1 (Supplementary Figure S3), indicating that all 21 species were under purifying selection.

Out of all the *ω* values calculated from the 13 mtDNA PCGs of the 21 examined mitogenomes, *atp8* had the highest average *ω* and *co1* had the lowest average *ω*. Thus, the *atp8* gene likely evolved more quickly, while the *co1* gene might have evolved more slowly, than the other mtDNA PCGs in the mitogenomes (Supplementary Figure S3).

The evolutionary rates of mtDNA PCGs in Gobiidae differed under different environmental selection pressures. The CmC model showed that *ω* < 1 in all mtDNA PCGs, indicating that these 13 genes were under purification-selection pressure during the evolution of Gobiidae (Table 5). The CmC model also showed that the nine mtDNA PCGs (except *co3*, *nd3*, *nd4*; *p* > 0.05) were significantly better than those in the M2a_rel model (*p* < 0.05). The *ω* values of three genes (*atp8*, *cyb*, *nd6*) in the EG group were higher than those in the SG and FG groups. The branch-site model showed that *nd6* and *atp8* genes had positively selected sites in the EG groups (Table 6).

## 4. Discussion

A common question in the field of evolutionary biology is how different environmental conditions shape mitogenomic evolution. Studies have revealed that organisms possess more patterns of adaptation to ecological change than expected [36]. In this comprehensive study, we presented a comparative analysis of the mitogenomes of Gobiidae species and examined natural selection at the molecular genetic level.

To the best of our knowledge, our study is the first to assemble the complete mitogenomes of *Chaenogobius annularis*, *Rhinogobius shennongensis*, and *Rhinogobius wuyanlingensis* using NOVOplasty software. The mitogenome structures and lengths (16,477–16,500 bp) of the three species were consistent with those of other fish species [8,37]. Similar to other animals [38,39], the mitogenomes of *Chaenogobius annularis*, *Rhinogobius shennongensis*, and *Rhinogobius wuyanlingensis* displayed typical circular structure, comprising 13 PCGs, 22 tRNA genes, two rRNA genes, and one partial CR (Figure 1 and Figure S1, Table 2, Table 3 and Table 4). The arrangement and orientation of the genes were similar to those found in other Gobiidae species determined in past research [40,41].

Our study identified some incomplete termination codons. Previous studies showed that the incomplete termination codon could be completed by posttranscriptional polyadenylation. *Rhiogobius shennongensis* and *Rhiogobius wuyanlingensis* belong to *Rhinogobius*, so the termination codons type is T for *co2*, *nd3*, *nd4*, and *cyb*, while the termination codon type is TA for *co3*. *Chaenogobius annularis* belongs to *Chaenogobius*. Based on the BI tree, this codon type has a distant developmental relationship with *Rhinogobius*; thus, T is the termination codon type of *co2*, *nd3*, and *nd4* in *Chaenogobius annularis*, while TA is the termination codon type of *co3*.

Previous studies showed that when observed ENc values exceed expected ENc values, complete mutational pressure occurs on the respective genes [42,43]. In contrast, when observed values are less than the expected values, selection pressure lowers the effective number of codons [44]. All plotted points in the current study were located below the umbrella line, indicating that there was more selection pressure than mutational bias on these genes. We also showed that all mitogenomes were translationally efficient, with natural selection playing an important role in these genomes.

Thirteen mtDNA PCGs in the mitochondria of all living organisms are considered important for ATP synthesis and heat generation [45]. Positive selection might provide important functional information relevant to adaptation to a new environment [46]. The ratio of *dN/dS* is an effective parameter for determining selection pressure [47]. To analyze pressure on mitochondrial PCGs, *dN/dS* ratios were evaluated for 21 species in our study. We found that each mtDNA PCG in the 21 species was generally lower than 1, indicating that mtDNA PCGs were subjected to purifying selection in different environments. Similar results were obtained by previous studies on marine turtles [36], horseshoe bats [48], and birds [49]. In addition, different *dN/dS* values for each of the 13 mtDNA PCGs indicate differing functional constraints among the genes [50]. Our results showed that difference in environments affects the evolutionary rate of mtDNA PCGs in Gobiidae. Our results also showed that *atp8* had a faster evolutionary rate than other mtDNA PCGs (Figure S1), indicating that *atp8* experienced more relaxed selective constraints than other mtDNA PCGs, allowing more mutations to accumulate [51].

The CmC model was used to explore whether different environments impacted Gobiidae. The *ω* ratios for the three branches (groups FG, SG, and EG) were far lower than 1. Thus, purifying selection was the major evolutionary pattern of mitochondria, implying strong evolutionary constraints on the mitogenome. This indicated the existence of strong selective pressure, with Gobiidae experiencing strong evolutionary constraints against the elimination of deleterious mutations and, hence, maintaining them. Previous studies recorded positive selection for *nd5* in vertebrates adapting to high altitudes [52]. Furthermore, positive selection was recorded for *nd4*, *cyb*, and *atp8* in the adaptation of bats to flight [53]. Our study showed that the genes *nd6* and *atp8* had positively selected sites in EG; thus, *nd6* and *atp8* might be essential for the evolutionary processes of EG. The peptides encoded by *nd6* were involved in the catalytic synthesis of ATP. Furthermore, *atp8* coded subunits of complex V of the respiratory chain, namely ATP synthase, which is responsible for ATP production [54,55]. The positive selection site signals for *nd6* and *atp8* found in EG might be relevant to their adaptations for energy requirements. The euryhaline group lives in multiple environments (i.e., along a varying saline gradient), and so requires more energy to adapt to associated changes; thus, positive gene selection sites might be associated with adaptation to energy metabolism in different environments. *Rhinogobius shennongensis*, *Rhinogobius wuyanlingensis*, and *Chaenogobius annularis* only inhabit freshwater and seawater, respectively, and so are subject to relatively less environmental pressure than the Euryhaline group. Thus, the evolution of mtDNA PCGs is key to organisms being able to adapt to different environments in Gobiidae. Furthermore, because of the large number of Gobiidae species and limited data, mitochondrial genome studies remain largely underexploited. We hope that our study will stimulate more genome studies on Gobiidae, and reveal more details on the molecular mechanisms underlying the adaptation of Gobiidae to different environments.

## 5. Conclusions

The fact that Gobiidae inhabit both freshwater and seawater shows that they have adapted to extremely harsh conditions, and are able to tolerate limited oxygen and high seawater salinity. Our study described the mitogenomes of *Chaenogobius annularis* (16,477 bp), *Rhinogobius shennongensis* (16,500 bp), and *Rhinogobius wuyanlingensis* (16,491 bp). These mitogenomes contained 13 PCGs, two rRNAs, 22 tRNAs, and one CR. We found that the *ω* ratios of all mtPCGs were <1, indicating that these genes perform an important function, and undergo purifying selection to maintain that function. The evolutionary rate of *atp8* in Gobiidae (all three environments) was higher than that of other mtDNA PCGs. We found evidence of positive selection at the coding level for several sites in the *nd6* and *atp8* genes for EG. This finding indicates an ability (EG group species) to adapt to differing energy requirements. In summary, our study provides molecular evidence for the evolution of different lifestyles, and valuable information for further phylogenetic and evolutionary research on the Gobiidae family.

## Figures and Tables

**Table 1:** Mitogenomes of Gobiidae species sequenced to date and their genomic features.

Species	Family	Accession Number	AT%	AT Skew	GC%	GC Skew	Length (bp)	Group
*Acanthogobius hasta*	*Gobiidae*	NC_006131.1	53.9	0.075	46.1	−0.256	16,663	seawater group (SG)
*Favonigobius gymnauchen*	*Gobiidae*	NC_047227.1	50.8	0.092	49.2	−0.269	16,480	
*Chaenogobius gulosus*	*Gobiidae*	NC_027193.1	59.0	0.113	41.0	−0.247	16,477	
*Rhinogobius giurinus*	*Gobiidae*	NC_022692.1	53.3	0.060	46.7	−0.311	16,520	
*Chaenogobius annularis*	*Gobiidae*	OM830225	58.9	0.145	41.1	−0.204	16,477	
*Mugilogobius chulae*	*Gobiidae*	NC_026519.1	54.4	0.081	45.6	−0.279	16,489	
*Amblychaeturichthys hexanema*	*Gobiidae*	NC_029228.1	54.9	0.093	45.1	−0.256	18,562	euryhaline group (EG)
*Chaeturichthys stigmatias*	*Gobiidae*	NC_020786.1	55.0	0.096	45.0	−0.254	18,562	
*Eucyclogobius newberryi*	*Gobiidae*	NC_028288.1	53.0	0.163	47.0	−0.226	16,523	
*Sicyopterus japonicus*	*Gobiidae*	NC_045074.1	53.7	0.050	46.3	−0.284	16,504	
*Gymnogobius petschiliensis*	*Gobiidae*	NC_008743.1	57.4	0.140	42.6	−0.228	16,424	
*Brachygobius doriae*	*Gobiidae*	NC_037142.1	60.8	0.049	39.2	−0.272	16,472	
*Rhinogobius cliffordpopei*	*Gobiidae*	NC_029252.1	49.4	0.097	50.6	−0.291	16,525	freshwater group (FG)
*Rhinogobius leavelli*	*Gobiidae*	NC_044964.1	51.8	0.080	48.2	−0.310	16,499	
*Rhinogobius rubromaculatus*	*Gobiidae*	NC_037144.1	51.5	0.089	48.5	−0.306	16,503	
*Mugilogobius myxodermus*	*Gobiidae*	NC_036070.1	54.2	0.082	45.8	−0.304	16,495	
*Schismatogobius ampluvinculus*	*Gobiidae*	NC_035717.1	52.3	0.098	47.7	−0.262	16,639	
*Sicyopus zosterophorus*	*Gobiidae*	NC_058982.1	55.0	0.057	45.0	−0.281	16,471	
*Micropercops swinhonis*	*Odontobutidae*	NC_021763.1	57.8	0.124	42.2	−0.225	16,493	
*Rhinogobius shennongensis*	*Gobiidae*	OM961050	52.3	0.044	47.7	−0.312	16,500	
*Rhinogobius wuyanlingensis*	*Gobiidae*	OM961051	53.2	0.070	46.8	−0.306	16,491	

**Table 2:** Characteristics of the mitochondrial genome of *Chaenogobius annularis*.

Gene	Nucleotide Positions	Size (bp)	Strand	Intergenic Nucleotide	Start	Stop
tRNA^PHE^	1–68	68	+			
12s rRNA	69–1014	946	+	0		
tRNA^VAL^	1015–1086	72	+	0		
16s rRNA	1091–2767	1677	+	4		
tRNA^LEU^	2768–2842	75	+	0		
*nd1*	2844–3818	975	+	1	ATG	TAA
tRNA^ILE^	3823–3893	71	+	4		
tRNA^GLN^	3894–3964	71	−	0		
tRNA^MET^	3964–4032	69	+	−1		
*nd2*	4033–5079	1065	+	0	ATG	TAA
tRNA^TRP^	5079–5150	72	+	−1		
tRNA^ALA^	5153–5221	69	−	2		
tRNA^ASN^	5223–5295	73	−	1		
tRNA^CYS^	5331–5395	65	−	35		
tRNA^TYR^	5396–5466	71	−	0		
*co1*	5468–7021	1554	+	1	GTG	TAA
tRNA^SER^	7022–7092	71	−	0		
tRNA^ASP^	7096–7167	72	+	3		
*co2*	7171–7861	691	+	3	ATG	T
tRNA^LYS^	7862–7936	75	+	0		
*atp8*	7938–8102	165	+	1	ATG	TAG
*atp6*	8096–8779	684	+	−7	ATG	TAA
*co3*	8779–9563	785	+	−1	ATG	TA
tRNA^GLY^	9563–9631	69	+	−1		
*nd3*	9632–9980	349	+	0	ATG	T
tRNA^ARG^	9981–10049	69	+	0		
*nd4l*	10050–10346	297	+	0	ATG	TAA
*nd4*	10340–11720	1381	+	−7	ATG	T
tRNA^HIS^	11721–11789	69	+	0		
tRNA^SER^	11790–11856	67	+	0		
tRNA^LEU^	11860–11932	73	+	3		
*nd5*	11933–13768	1836	+	0	ATG	TAG
*nd6*	13765–14286	522	−	−4	ATG	TAA
tRNA^GLU^	14287–14355	69	−	0		
*cyb*	14361–15498	1138	+	5	ATG	TAG
tRNA^THR^	15502–15573	72	+	3		
tRNA^PRO^	15574–15643	70	−	0		

**Table 3:** Characteristics of the mitochondrial genome of *Rhinogobius shennongensis*.

Gene	Nucleotide Positions	Size (bp)	Strand	Intergenic Nucleotide	Start	Stop
tRNA^PHE^	1–68	68	+			
12s rRNA	69–1017	949	+	0		
tRNA^VAL^	1018–1089	72	+	0		
16s rRNA	1098–2778	1681	+	8		
tRNA^LEU^	2779–2853	75	+	0		
*nd1*	2854–3828	975	+	0	ATG	TAA
tRNA^ILE^	3831–3900	70	+	2		
tRNA^GLN^	3900–3970	71	−	−1		
tRNA^MET^	3970–4038	69	+	−1		
*nd2*	4039–5085	1047	+	0	ATG	TAA
tRNA^TRP^	5087–5157	71	+	1		
tRNA^ALA^	5160–5228	69	−	2		
tRNA^ASN^	5230–5302	73	−	1		
tRNA^CYS^	5335–5399	65	−	32		
tRNA^TYR^	5400–5469	70	−	0		
*co1*	5471–7024	1554	+	1	GTG	TAA
tRNA^SER^	7025–7095	71	−	0		
tRNA^ASP^	7099–7170	72	+	3		
*co2*	7174–7864	691	+	3	ATG	T
tRNA^LYS^	7865–7940	76	+	0		
*atp8*	7942–8106	165	+	1	ATG	TAA
*atp6*	8100–8783	684	+	−7	ATG	TAA
*co3*	8783–9567	785	+	−1	ATG	TA
tRNA^GLY^	9567–9638	72	+	−1		
*nd3*	9639–9987	349	+	0	ATG	T
tRNA^ARG^	9988–10,056	69	+	0		
*nd4l*	10,057–10,353	297	+	0	ATG	TAA
*nd4*	10,347–11,727	1381	+	−7	ATG	T
tRNA^HIS^	11,728–11,796	69	+	0		
tRNA^SER^	11,797–11,864	68	+	0		
tRNA^LEU^	11,869–11,941	73	+	4		
*nd5*	11,942–13,780	1839	+	−4	ATG	TAA
*nd6*	13,777–14,298	522	−	−4	ATG	TAG
tRNA^GLU^	14,299–14,367	69	−	0		
*cyb*	14,373–15,512	1140	+	5	ATG	T
tRNA^THR^	15,514–15,585	72	+	1		
tRNA^PRO^	15,585–15,645	61	−	−1		

**Table 4:** Characteristics of the mitochondrial genome of *Rhinogobius wuyanlingensis*.

Gene	Nucleotide Positions	Size (bp)	Strand	Intergenic Nucleotide	Start	Stop
tRNA^PHE^	1–68	68	+			
12s rRNA	69–1019	951	+	0		
tRNA^VAL^	1019–1090	72	+	−1		
16s rRNA	1099–2774	1676	+	8		
tRNA^LEU^	2775–2848	74	+	0		
*nd1*	2849–3823	975	+	0	ATG	TAA
tRNA^ILE^	3828–3897	70	+	4		
tRNA^GLN^	3897–3967	71	−	−1		
tRNA^MET^	3967–4035	69	+	−1		
*nd2*	4036–5082	1047	+	0	ATG	TAA
tRNA^TRP^	5084–5154	71	+	1		
tRNA^ALA^	5157–5225	69	−	2		
tRNA^ASN^	5227–5299	73	−	1		
tRNA^CYS^	5332–5397	66	−	2		
tRNA^TYR^	5398–5467	70	−	0		
*co1*	5469–7022	1554	+	1	GTG	TAA
tRNA^SER^	7023–7093	71	−	0		
tRNA^ASP^	7097–7168	72	+	3		
*co2*	7172–7862	691	+	3	ATG	T
tRNA^LYS^	7863–7938	76	+	0		
*atp8*	7940–8104	165	+	1	ATG	TAA
*atp6*	8098–8781	684	+	−7	ATG	TAA
*co3*	8781–9565	785	+	−1	ATG	TA
tRNA^GLY^	9565–9635	71	+	−1		
*nd3*	9636–9984	349	+	0	ATG	T
tRNA^ARG^	9985–10,053	69	+	0		
*nd4l*	10,054–10,350	297	+	0	ATG	TAA
*nd4*	10,344–11,724	1381	+	−7	ATG	T
tRNA^HIS^	11,725–11,792	68	+	0		
tRNA^SER^	11,793–11,860	68	+	0		
tRNA^LEU^	11,865–11,937	73	+	4		
*nd5*	11,938–13,776	1839	+	0	ATG	TAG
*nd6*	13,773–14,294	522	−	−4	ATG	TAG
tRNA^GLU^	14,295–14,363	69	−	0		
*cyb*	14,369–15,508	1140	+	5	ATG	T
tRNA^THR^	15,510–15,581	72	+	1		
tRNA^PRO^	15,581–15,650	139	−	−1		

**Table 5:** Test for positive selection in divergent clades of each mtDNA PCG using Clade model C.

Gene	Model Compared	|2ΔlnL|	*p*-Value	*ω*_SG_	*ω*_EG_	*ω*_FG_
*atp6*	M2a_rel vs. CmC	10.16444	0.017218814 *	0.13766	0.06356	0.08155
*atp8*		28.09846	3.46325×10^-6^ **	0.30112	0.36634	0.11016
*co1*		123.17652	1.59679×10^-26^ **	0.10628	0.13944	0.15688
*co2*		15.34283	0.001545921 **	0.07167	0.04816	0.06300
*co3*		3.31304	0.34583	0.09589	0.09700	0.10508
*cyb*		234.47697	1.48869×10^-50^ **	0.06100	0.10580	0.06614
*nd1*		8.34667	0.039364856 *	0.10092	0.04505	0.13131
*nd2*		3.35000	0.000227486 **	0.10087	0.07880	0.11073
*nd3*		5.49261	0.13908	0.13504	0.07199	0.14217
*nd4*		3.93641	0.26841	0.12748	0.10171	0.13955
*nd4l*		8.604689	0.035035806 *	0.08711	0.06757	0.08709
*nd5*		271.95414	1.16596×10^-58^ **	0.12279	0.11736	0.15599
*nd6*		1407.00957	8.8747×10^-305^ **	0.07906	0.22650	0.15444

Note: * Significant level (** *p* < 0.01, * *p* < 0.05). M2a_rel: null model; CmC: Clade model C; |2ΔlnL| is the log-likelihood score; *ω* is the evolution rate. FG: freshwater group; SG: seawater group; EG: euryhaline group.

**Table 6:** Positive selection for 13 mtDNA PCGs in the freshwater, seawater, and euryhaline groups based on the branch-site model.

Gene	Model	2ΔLNL	*p*-Value	Positively Selected Sites (BEB Analysis)	Foreground Branch	Background Branch
*nd6*	Model A Null Model	0	1	2 S 0.963 *; 100 G 0.977 *; 139 F 0.989 *	EG	FG and SG
*atp8*		0	1	7 S 0.999 **; 25 V 0.988 *; 36 E 0.978 *; 41 N 1.000 **; 51 S 0.966 *	EG	FG and SG

Note: BEB analysis: Bayes empirical Bayes analysis; * Significant level (** *p* < 0.01, * *p* < 0.05). FG: freshwater group; SG: seawater group; EG: euryhaline group.

## Data Availability

All the mitochondria genomes sequences used in this study were accessed through the GenBank database using the accession numbers in Table 1.

## Supplementary Materials

The following supporting information can be downloaded at: www.mdpi.com/article/10.3390/ani12141741/s1, Supplementary Figure S1: Gene map of mitogenome of *Chaenogobius annularis* (a), *Rhinogobius wuyanlingensis* (b). The genes outside the circle were transcribed clockwise, while the genes inside were transcribed counterclockwise. Supplementary Figure S2: The relative synonymous codon usage (RSCU) of *Chaenogobius annularis* (a), *Rhinogobius wuyanlingensis* (b). Codon families are plotted on the X axis. Supplementary Figure S3: Boxplot of *dN/dS* (*ω*) value of the 13 mtDNA PCGs (each mtDNA PCG) across the 21 species mitogenomes; Table S1: Positive selection on 13 mtDNA PCGs of euryhaline group (foreground branch), seawater group and freshwater group (background branch) through branch-site model. Supplementary Table S2: The ω values of 13 mtDNA PCGs and each mtDNA PCGs of 21 species.

## References

[1] Parenti L.R., Nelson J.S. (1995). Fishes of the World. Copeia.

[2] Hubbs C., Moser H.G., Richards W.J., Cohen D.M., Fahay M.P., Kendall A.W., Richardson S.L. (1985). Ontogeny and Systematics of Fishes. Copeia.

[3] Bauchot M.L., Hureau J.C. (1986). Fishes of the North-Eastern Atlantic and the Mediterranean. Unesco.

[4] Van der Giezen M., Tovar J., Clark C.G. (2005). Mitochondrion-Derived Organelles in Protists and Fungi. Int. Rev. Cytol..

[5] Van der Bliek A.M., Sedensky M.M., Morgan P.G. (2017). Cell Biology of the Mitochondrion. Genetics.

[6] Brand M.D. (1997). Regulation analysis of energy metabolism. J. Exp. Biol..

[7] Shinde S., Bhadra U. (2015). A complex genome-microRNA interplay in human mitochondria. BioMed Res. Int..

[8] Liu Y., Wu P.D., Zhang D.Z., Zhang H.B., Tang B.P., Liu Q.N., Dai L.S. (2019). Mitochondrial genome of the yellow catfish *Pelteobagrus fulvidraco* and insights into Bagridae phylogenetics. Genomics.

[9] Mascolo C., Ceruso M., Palma G., Anastasio A., Pepe T., Sordino P. (2018). The complete mitochondrial genome of the Pink dentex *Dentex gibbosus* (Perciformes: Sparidae). Mitochondrial DNA Part B Resour..

[10] Taanman J.-W. (1999). The mitochondrial genome: Structure, transcription, translation and replication. Biochim. Biophys. Acta (BBA)-Bioenerg..

[11] Yang C., Chen Y., Chen Z., He G., Zhong Z., Xue W. (2020). The next-generation sequencing reveals the complete mitochondrial genome of *Rhinogobius formosanus* (Perciformes: Gobiidae). Mitochondrial DNA Part B Resour..

[12] Dhar D., Dey D., Basu S., Fortunato H. (2021). Insight into the adaptive evolution of mitochondrial genomes in intertidal chitons. J. Molluscan Stud..

[13] Sokolova I. (2018). Mitochondrial Adaptations to Variable Environments and Their Role in Animals’ Stress Tolerance. Integr. Comp. Biol..

[14] Lartigue L., Faustin B. (2013). Mitochondria: Metabolic regulators of innate immune responses to pathogens and cell stress. Int. J. Biochem. Cell Biol..

[15] Caccone A., Gentile G., Burns C.E., Sezzi E., Bergman W., Ruelle M., Saltonstall K., Powell J.R. (2004). Extreme difference in rate of mitochondrial and nuclear DNA evolution in a large ectotherm, Galapagos tortoises. Mol. Phylogenetics Evol..

[16] Harrison R.G. (1989). Animal mitochondrial DNA as a genetic marker in population and evolutionary biology. Trends Ecol. Evol..

[17] Gissi C., Iannelli F., Pesole G. (2008). Evolution of the mitochondrial genome of Metazoa as exemplified by comparison of congeneric species. Heredity.

[18] Saccone C., De Giorgi C., Gissi C., Pesole G., Reyes A. (1999). Evolutionary genomics in Metazoa: The mitochondrial DNA as a model system. Gene.

[19] Lopez-Lopez A., Vogler A.P. (2017). The mitogenome phylogeny of Adephaga (Coleoptera). Mol. Phylogenetics Evol..

[20] Liu M., Zhang Z., Peng Z. (2015). The mitochondrial genome of the water spiderArgyroneta aquatica(Araneae: Cybaeidae). Zool. Scr..

[21] Zhang Z., Xing Y., Cheng J., Pan D., Lv L., Cumberlidge N., Sun H. (2020). Phylogenetic implications of mitogenome rearrangements in East Asian potamiscine freshwater crabs (Brachyura: Potamidae). Mol. Phylogenetics Evol..

[22] Whitehead A. (2009). Comparative mitochondrial genomics within and among species of killifish. BMC Evol. Biol..

[23] Li F., Lv Y., Wen Z., Bian C., Zhang X., Guo S., Shi Q., Li D. (2021). The complete mitochondrial genome of the intertidal spider (*Desis jiaxiangi*) provides novel insights into the adaptive evolution of the mitogenome and the evolution of spiders. BMC Ecol. Evol..

[24] Dierckxsens N., Mardulyn P., Smits G. (2017). NOVOPlasty: De novo assembly of organelle genomes from whole genome data. Nucleic Acids Res..

[25] Bernt M., Donath A., Juhling F., Externbrink F., Florentz C., Fritzsch G., Putz J., Middendorf M., Stadler P.F. (2013). MITOS: Improved de novo metazoan mitochondrial genome annotation. Mol. Phylogenetics Evol..

[26] Kumar S., Stecher G., Li M., Knyaz C., Tamura K., Battistuzzi F.U. (2018). MEGA X: Molecular Evolutionary Genetics Analysis across Computing Platforms. Mol. Biol. Evol..

[27] He W., Wang N., Tan J., Wang R., Yang Y., Li G., Guan H., Zheng Y., Shi X., Ye R. (2019). Comprehensive codon usage analysis of porcine deltacoronavirus. Mol. Phylogenetics Evol..

[28] Perna N.T., Kocher T.D. (1995). Patterns of nucleotide composition at fourfold degenerate sites of animal mitochondrial genomes. J. Mol. Evol..

[29] Edgar R.C. (2004). MUSCLE: Multiple sequence alignment with high accuracy and high throughput. Nucleic Acids Res..

[30] Zhang D., Gao F., Jakovlić I., Zou H., Zhang J., Li W.X., Wang G.T. (2020). PhyloSuite: An integrated and scalable desktop platform for streamlined molecular sequence data management and evolutionary phylogenetics studies. Mol. Ecol. Resour..

[31] Ronquist F., Teslenko M., van der Mark P., Ayres D.L., Darling A., Hohna S., Larget B., Liu L., Suchard M.A., Huelsenbeck J.P. (2012). MrBayes 3.2: Efficient Bayesian phylogenetic inference and model choice across a large model space. Syst. Biol..

[32] Letunic I., Bork P. (2019). Interactive Tree Of Life (iTOL) v4: Recent updates and new developments. Nucleic Acids Res..

[33] Yang Z. (2007). PAML 4: Phylogenetic analysis by maximum likelihood. Mol. Biol. Evol..

[34] Wang H.Y., Tsai M.P., Dean J., Lee S.C. (2001). Molecular phylogeny of gobioid fishes (Perciformes: Gobioidei) based on mitochondrial 12S rRNA sequences. Mol. Phylogenetics Evol..

[35] Maxfield J.M., Van Tassell J.L., St Mary C.M., Joyeux J.C., Crow K.D. (2012). Extreme gender flexibility: Using a phylogenetic framework to infer the evolution of variation in sex allocation, phylogeography, and speciation in a genus of bidirectional sex changing fishes(Lythrypnus, Gobiidae). Mol. Phylogenetics Evol..

[36] Ramos E., Freitas L., Nery M.F. (2020). The role of selection in the evolution of marine turtles mitogenomes. Sci. Rep..

[37] Zhang Y., Li X.H., Tian F., Liu S.J., Feng C.G., Zhao K. (2020). Mitochondrial genome and phylogenetic relationship of *Gymnocypris eckloni* (Schizothoracinae) in Qaidam river basin. Genomics.

[38] Yi J., Wu H., Liu J., Li J., Lu Y., Zhang Y., Cheng Y., Guo Y., Li D., An Y. (2022). Novel gene rearrangement in the mitochondrial genome of *Anastatus fulloi* (Hymenoptera Chalcidoidea) and phylogenetic implications for Chalcidoidea. Sci. Rep..

[39] Sun Y., Huang H., Liu Y., Liu S., Xia J., Zhang K., Geng J. (2021). Organization and phylogenetic relationships of the mitochondrial genomes of Speiredonia retorta and other lepidopteran insects. Sci. Rep..

[40] Kim I.C., Kweon H.S., Kim Y.J., Kim C.B., Gye M.C., Lee W.O., Lee Y.S., Lee J.S. (2004). The complete mitochondrial genome of the javeline goby *Acanthogobius hasta* (Perciformes, Gobiidae) and phylogenetic considerations. Gene.

[41] Liu T., Jin X., Wang R., Xu T. (2013). Complete sequence of the mitochondrial genome of *Odontamblyopus rubicundus* (Perciformes: Gobiidae): Genome characterization and phylogenetic analysis. J. Genet..

[42] Roy A., Mukhopadhyay S., Sarkar I., Sen A. (2015). Comparative investigation of the various determinants that influence the codon and amino acid usage patterns in the genus Bifidobacterium. World J. Microbiol. Biotechnol..

[43] Smith R. (2019). Enhanced effective codon numbers to understand codon usage bias. BioRxiv.

[44] Dey P., Sharma S.K., Sarkar I., Ray S.D., Pramod P., Kochiganti V.H.S., Quadros G., Rathore S.S., Singh V., Singh R.P. (2021). Complete mitogenome of endemic plum-headed parakeet Psittacula cyanocephala-characterization and phylogenetic analysis. PLoS ONE.

[45] Wang Y., Shen Y., Feng C., Zhao K., Song Z., Zhang Y., Yang L., He S. (2016). Mitogenomic perspectives on the origin of Tibetan loaches and their adaptation to high altitude. Sci. Rep..

[46] Nielsen R. (2005). Molecular signatures of natural selection. Annu. Rev. Genet..

[47] Yang Z., Bielawski J.P. (2000). Statistical methods for detecting molecular adaptation. Trends Ecol. Evol..

[48] Zhang L., Sun K., Csorba G., Hughes A.C., Jin L., Xiao Y., Feng J. (2021). Complete mitochondrial genomes reveal robust phylogenetic signals and evidence of positive selection in horseshoe bats. BMC Ecol. Evol..

[49] Shen Y.Y., Shi P., Sun Y.B., Zhang Y.P. (2009). Relaxation of selective constraints on avian mitochondrial DNA following the degeneration of flight ability. Genome Res..

[50] Muse S.V. (2000). Examining rates and patterns of nucleotide substitution in plants. Plant Mol. Biol..

[51] Yang H., Li T., Dang K., Bu W. (2018). Compositional and mutational rate heterogeneity in mitochondrial genomes and its effect on the phylogenetic inferences of Cimicomorpha (Hemiptera: Heteroptera). BMC Genom..

[52] Wang X., Zhou S., Wu X., Wei Q., Shang Y., Sun G., Mei X., Dong Y., Sha W., Zhang H. (2021). High-altitude adaptation in vertebrates as revealed by mitochondrial genome analyses. Ecol. Evol..

[53] Shen Y.Y., Liang L., Zhu Z.H., Zhou W.P., Irwin D.M., Zhang Y.P. (2010). Adaptive evolution of energy metabolism genes and the origin of flight in bats. Proc. Natl. Acad. Sci. USA.

[54] Anderson S., Bankier A.T., Barrell B.G., de Bruijn M.H., Coulson A.R., Drouin J., Eperon I.C., Nierlich D.P., Roe B.A., Sanger F. (1981). Sequence and organization of the human mitochondrial genome. Nature.

[55] Jonckheere A.I., Smeitink J.A., Rodenburg R.J. (2012). Mitochondrial ATP synthase: Architecture, function and pathology. J. Inherit. Metab. Dis..

