# Supplementary material for: Adaptability and Evolution of Gobiidae: A Genetic Exploration
Source: Animals (Basel). 2022 Jul 6;12(14):1741. doi: 10.3390/ani12141741 (PMC9312210; doi:10.3390/ani12141741)
Supplement: Supplementary file 1 [file animals-12-01741-s001.zip › Supplementary_Material.pdf]

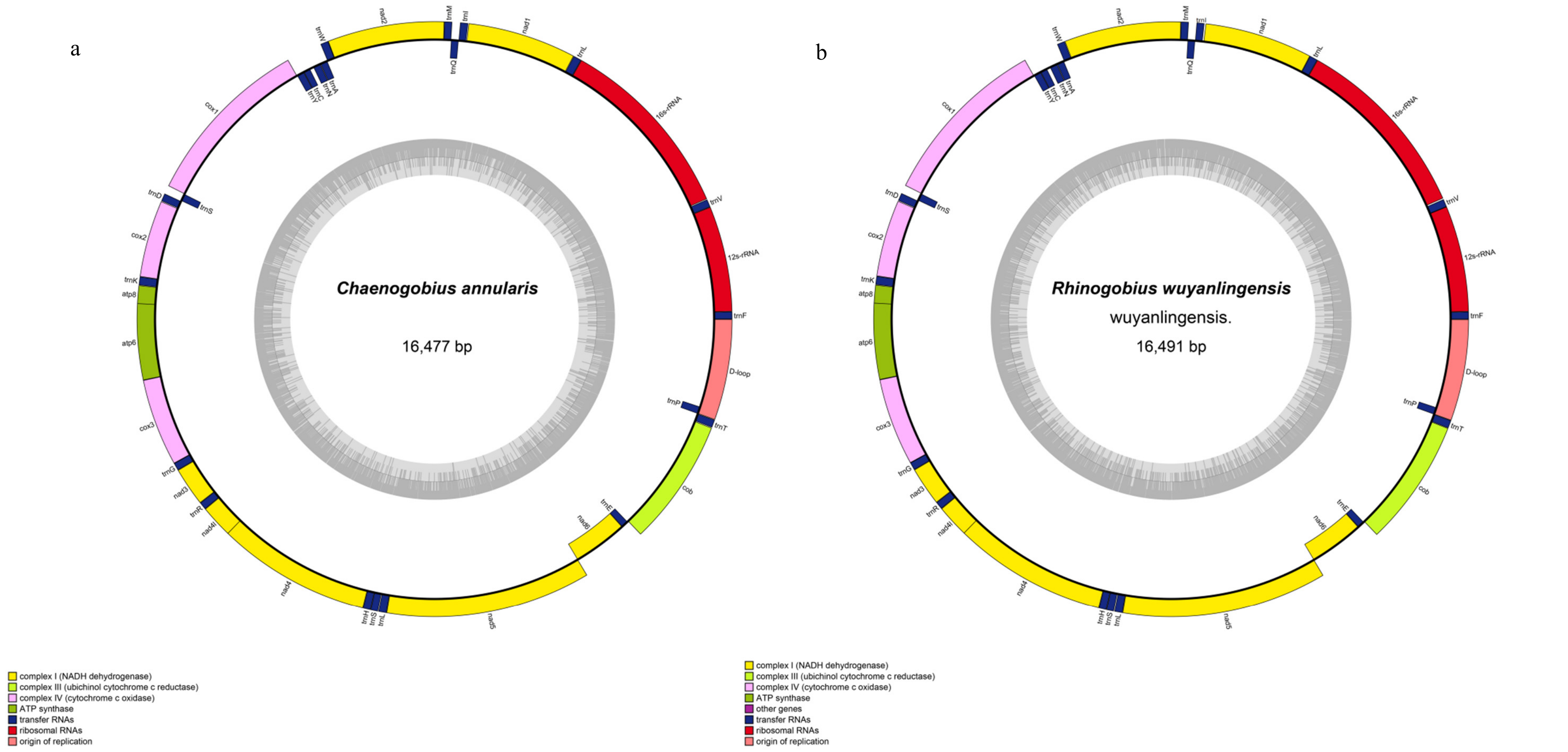

Supplementary Figure S1. Gene map of mitogenome of *Chaenogobius annularis* (a), *Rhinogobius wuyanlingensis* (b). The genes outside the circle were transcribed clockwise, while the genes inside were transcribed counterclockwise.

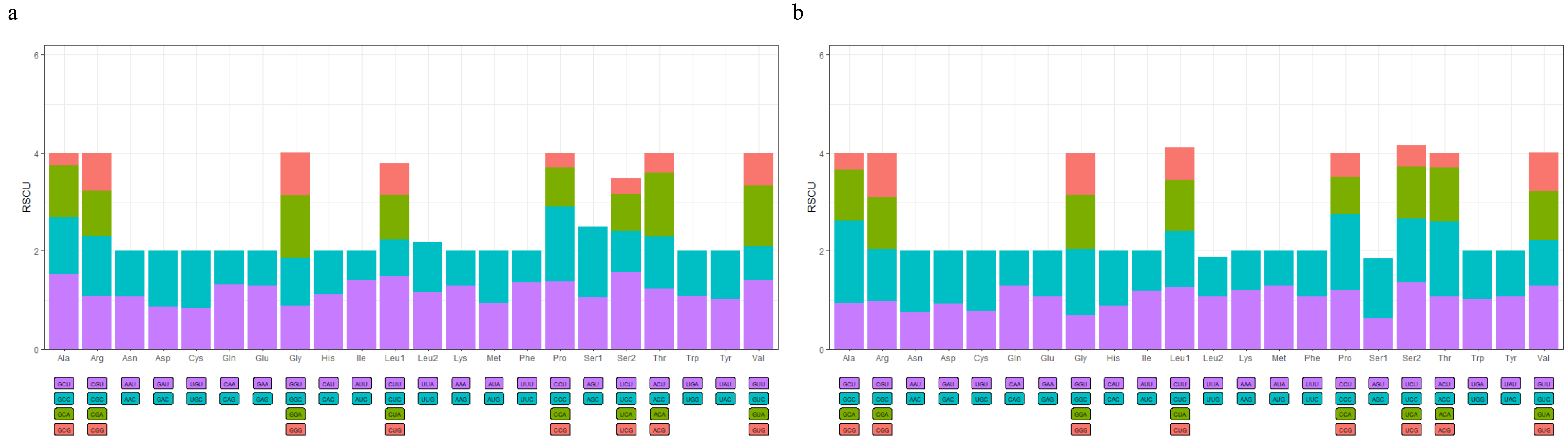

Supplementary Figure S2. The relative synonymous codon usage (RSCU) of *Chaenogobius annularis* (a), *Rhinogobius wuyanlingensis* (b). Codon families are plotted on the X axis.

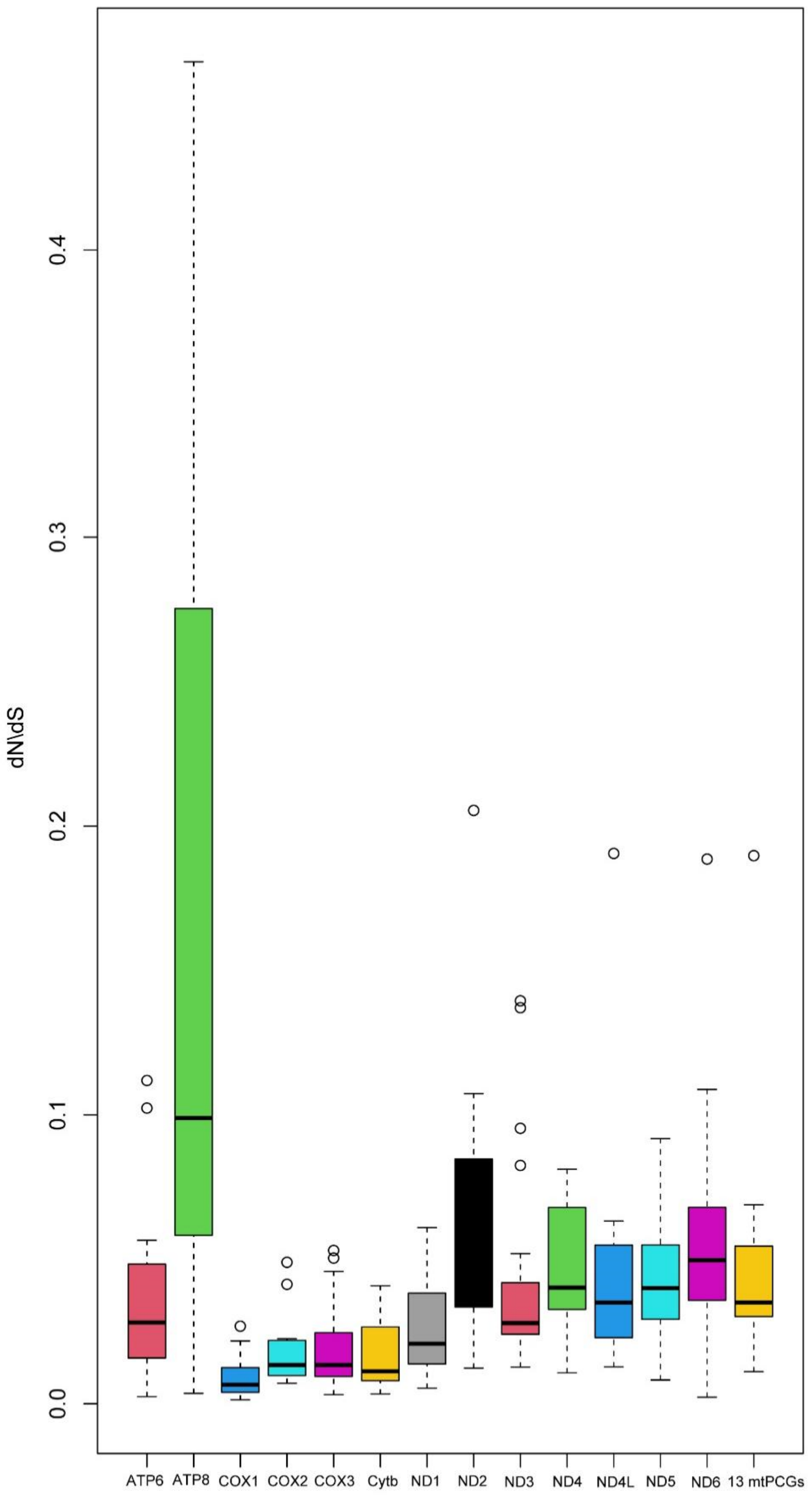

Supplementary Figure S3. Boxplot of dN/dS (ω) value of the 13 mtDNA PCGs (each mtDNA PCG) across the 21 species mitogenomes.

Table S1 Positive selection on 13 mtDNA PCGs of euryhaline group (foreground branch), seawater group and freshwater group (background branch) through branch-site model.

Gene	Model	2△LNL	p-value	Positively selected sites (BEB analysis)	Foreground branch	Background branch
ATP6		2×10^{-6}	0.998871621	13 V 0.568; 68 K 0.592		
ATP8		0	1	7 S 0.999**; 25 V 0.988*; 36 E 0.978*; 41 N 1.000**; 51 S 0.966*		
COX1		0.040278	0.840938011			
COX2		0	1			
COX3		0	1			
CYTB		9.315194	0.002272613			
ND1		0	1	323 L 0.556		
ND2	Model A	0	1	88 L 0.547; 205 T 0.725	EG	FG and SG
ND3	Null Model	0	1			
ND4		0	1	26 A 0.801; 344 V 0.835		
ND4L		0	1			
ND5		0	1	38 L 0.866; 77 S 0.994**; 121 R 0.651; 209 T 0.652; 210 A 0.968*; 268 M 0.998**; 490 L 0.660;b 581 T 0.909		
ND6		0	1	2 S 0.963*; 100 G 0.977*; 139 F 0.989*		

Note: BEB analysis: Bayes empirical Bayes analysis; * Significant level (**$p < 0.01$, *$p < 0.05$). FG: freshwater group; SG: seawater group; EG: euryhaline group.
